# Foreign Correspondence

**Published:** 1872-02

**Authors:** A. W. Calhoun

**Affiliations:** Berlin, Prussia


### Foreign Correspondence.

### CHRONIC OTORRHŒA AND ITS CURE BY ALCOHOL.

BY A. W. CALHOUN, M. D., (NOW) OF BERLIN, PRUSSIA.

A disease, coming so often before the eye of the physician, and whose treatment, in general, is so very unsatisfactory, both to patient and medical adviser, demands careful and especial inquiry. Such, I have now under consideration in Chronic Otorrhœa, and in proof of its obstinacy, and the ill success of its management, is the fact, that we almost daily see individuals of all ages, bearing offensive evidences of its existence, and who tell us that, notwithstanding the treatment extending throught a series of years, the disease has steadily progressed from childhood. The consequences of this affection, are not generally enough known, and therefore, is the disease not actively enough attacked. Many children have died, while suffering from Chronic Purulent Otitis, from "Brain Trouble," which was supposed to have depended upon causes, entirely foreign to the real ones, when, upon a post-mortem examination being performd, it was found that the inflammation had extended from the deeper structures of the ear into the surrounding bases, and from thence, into the membranes of the brain, producing "meningitis," and in some cases, even abscess in the brain substance. A knowledge of the anatomy of the ear and the relation of its parts to the base of the brain, will easily demonstrate how these results can follow.

It is not my intention to enter upon the causes of Otorrhœa, or of its beginning symptoms—but to speak of it, after it has made itself known, by an open discharge. So, a few general remarks upon this point, will suffice for my object.

Occasionally the disease is situtated in the external meatus, without any complication with other diseased structures—and is then, simply an inflammation of the membrane, lining this canal. But, in a large majority of cases, it is a trouble of the middle ear, involving the bones contained therein, and the "membrana tympani." Indeed, where the fœtid discharge has continued for six months, or one or two years, it has, almost invariably, been found that perforation of the Mem. Tymp. existed, with the mucous membrane, lining the cavity, in a highly inflamed state. Frequently, also, naso-pharyngitis, is a concomitant symptom, which assists in keeping up a diseased condition, by extension of the inflammation into the middle ear, through the eustachian tube. This should be carefully attended to, in the treatment of all cases.

Where polypoid growths, or caries, are present, they require remedies peculiar to themselves—when the co-existing Otorrhœa will pass away, in all probability, with a removal of its cause.

I had heard much, and had read several articles in the medical journals of Berlin, in praise of the use of pure, undiluted alcohol, for the cure of uncomplicated Chronic Purulent Otitis, or Otorrhœa, as practiced in the Clinic of Dr. Weber, one of the editors of the "Monatschrift fur Ohrenheilkunde," and must confess, that I was somewhat sceptical, as to the propriety of its use—believing, that so strong a medicine, upon so delicate a structure, as that covering the interior of the cavity of the tympanum, would be greater source of harm than good. Through the kindness of Dr. W., I have been enabled to follow quite a number of cases, as they progressed under this treatment, from the date of their first entrance into the Clinic, till their discharge, as cured—and am now, thoroughly satisfied of its efficacy, having seen cases of eight and ten years standing, with important destruction of Mem. Tymp., cured in comparatively short time. Of course, large perforations of the Mem. Tymp., having continued for a long while, are never filled by new-formed membranes, but remain always open. With the structures in the interior of the cavity, in tolerable condition, even though a moderate sized perforation *does* continue to exist, the hearing is generally quite good.

One would suppose, that the application of the undiluted alcohol upon *any* of the *sound* mucous membranes, would produce intense pain, and much more so, upon the inflamed membrane in the cavity of the tympanum. But such is not the case—for a few moments, there is a smarting, slightly burning feeling, which gives place to a sensation, described by patients, as that of an "agreeable warmness." This latter, lasts for a considerable time.

These polyclinics are held once a week, exclusively for the poor, and in general, a majority of the patients are in bad condition for any and every treatment. They come, also, in such numbers, that there is not sufficient time to give to each case, strict or especial consideration—but, as a rule, these Germans are quite apt, and carry out the half-caught ideas, much to the benefit of their sufferings.

It is best not to recommend the general use of milk-warm water,

(as is usually done) in syringing and cleansing the ear, as a majority of the common people—among whom, this disease prevails more generally—fail to thoroughly dry the interior, which is of the greatest importance, since, by a few drops of water remaining behind, for some time, an acute catarrh is frequently produced, in addition to the already existing disease, which certainly makes matters worse. But to cleanse the ear, with a fine Camel's hair pencil, each time before using the alcohol. When the physician is present, the syringe can, of course, be used, and he can afterwards, attend himself to the drying of the parts, but direct the patients, at home, to use the pencil. When the patient presents himself at the Clinic, the "Air Douche" is first given, which consists in pumping air into the middle ear, through a catheter, introduced into the eustachian tube, for the purpose of opening this tube, and freeing it of pus. Then the ear is cleansed, in the manner described. Dr. Weber very seldom uses the syringe—only the hair pencil. Rest the head to one side, upon a table for instance, and completely fill the external meatus with the alcohol. Allow the head to remain in this position for about 5 minutes, rubbing and pressing gently upon the "Tragus," so that the fluid may come in contact with, and pass into the eustachian tube. Frequently, a few drops will pass through the tube into the mouth. As before remarked, the pain is slight and but momentary, when a pleasant feeling ensues. Keep the ear filled for the above length of time, then turn the alcohol out, and fill the meatus with charpie or lint, to the extent of keeping out all air. Direct the patient to proceed thus, three times daily, till there is a well-observed diminution in the discharge—afterward twice daily, till the disease has disappeared. For a while, after its disappearance, it is prudent to continue the medicine once daily.

Enjoin strict abstinence from all stimulants, direct patients to avoid taking fresh colds, and to keep the bowels in good condition.

Where constitutional symptoms accompany the Otorrhœa, constitutional treatment is also required. If granulations exist, they must first be treated by solutions of "sulph. copper," or "nitrate of silver," and after their removal, resort, if necessary, to the alcohol. Should "Eczema" be combined with the discharge, use in connection with the spiritus, a small quantity of corrosive sublimate, $\frac{1}{8}$ to $\frac{1}{4}$ gr. to the oz.

The theory of the action of this medicine is, that it destroys and keeps in this condition the animalculæ, which are generated upon the diseased surfaces and in the pus, by contact with the atmosphere; and the destruction of these animalculæ being accomplished, the disease passes away almost of its own accord, since upon them, it, to a great extent, depends.

The simplicity, reliability and success of this treatment, when properly carried out, upon proper cases, recommends itself to every physician who has to do with such sufferings.

Below are a few cases showing the rapid healing of this affection, treated *solely* by alcohol. I have quite a number upon my note-book, but believe these will be sufficient for demonstration—and hope the success here pictured, may induce some who read this, to at least endeavor, to relieve the scores in their midst, who go through life an annoyance to themselves and friends, and shunned by society :

*Case* 1. O. L——, man, æt 17. 5 years ago had quite a servere attack of catarrhal affection, at which time he lost, to a great extent, "hearing" in both ears, and which has continued till now, with only occasional improvement, to be again renewed upon every fresh attack of "cold." Came to this Clinic, once or twice, in 1870; not since, till to-day, Oct. 1st, 1871. Complains now of much pain in the region of, and in the ears, great deafness and a most offensive discharge from both sides. Exploration shows perforation of "membrana tympani" in each ear, exposure of the cavities, with intense inflammation of the same and their contents, and a most copius secretion, both from here and the "external meatus." Bones of the middle ear much inflamed, but not necrosed! Cleansed the ears with a fine Camel's-hair pencil, and used "Air Douche," in the manner before described. Applied alcohol to the parts and allowed it to remain some minutes in contact with the diseased surfaces; filled the meatus with charpie, to the extent of keeping out all air, and prescribed the use of the same remedies three times daily, at home.

October 15th—Discharge much less, and with but little of its former offensiveness. Cavity of tympanum healing, hearing better. Used "Air Douche," and continued the use of the alcohol.

October 29th—Perforation of right membrane entirely healed, the left somewhat smaller, the discharge and hearing still improving, continued the same treatment.

November 5th—Secretion has entirely ceased, slight cicatrices extending across both membranes, hears now, the ticking of a watch several feet distant, whereas, three or four weeks ago, it could not be heard two or three inches. No pain—discharged as cured.

*Case* 2.—October 20th, A. B——, girl, æt 11 years. This little girl is of a scrofulous diathesis—badly nourished—is now quite emaciated, and, in fact, one of the worst cases for treatment, since all of her surroundings are bad. Has had chronic Otorrhœa in both ears since 5 years of age, and has been treated variously, by different physicians, with no apparent good result. Came first to Clinic two months ago, and has presented herself regularly, since then, once a week, and been treated only by the "Air Douche" and alcohol, and used the latter at home, three times daily, with the necessary precautions for cleanliness. She has lived upon a nourishing diet, as her weakened condition demanded. The Otorrhœa has disappeared, health much improved, and she presents quite a vigorous appearance to that of a few weeks ago. No further treatment necessary for the *ear trouble, but to continue, for a while, the strong diet.*

*Case* 3.—J. L——, man, æt 25 years, presented himself August 15th, with deafness, and a most profuse and almost sickening discharge from the cavity of right tympanum, through a large perforation of the membrane. This state of things has continued for several years. Could not, at the above time, hear any considerable noise, even one foot distant, with this ear. Being a servant, could not attend the Clinic oftener than once every two weeks, when the "Air Douche" and alcohol were used, and patient directed to follow up the same at home, three times daily.

October 29th—Otorrhœa quite gone, and also, all inflammatory symptoms. Hears now, with ease, at 8 feet. The hearing in this case, cannot be further improved (except by artificial means), since the larger portion of the "membrana tympani," as well as a part of the bones occupying the cavity, have been destroyed by the previous inflammation. Here, that peculiar sound, called "perforation *noise*," charateristic of an opening through the membrane, is plainly heard, upon air being thrown into the cavity, through the eustachian tube. Patient discharged as well, but directed to continue the use of the *alcohol for a short time, for prudence sake.*

*Case* 4.—S. D——, boy, æt 9 years· When 1 year old, the disease began, and has continued up to the present time. Perforations in both ears, and a very free and fœtid secretion, hears only moderately well, and at a short distance. Came first to the Clinic at the beginning of August, when he was treated with the local application of alcohol and Air Douche, and absolute cleanliness of the diseased parts enjoined. This has been practiced in this case, once each week, in the Clinic, and the alcohol used in the interval, at home, three times daily.

October 29th.—Patient returns to-day with no discharge, all inflammation has disappeared. One membrane healed over, the other remains with perforation, but well, hearing much improved. To continue, for a short time, the use of the alcohol, once daily.

November 5th—*Patient completely cured and discharged.*

*Case* 5.—T. S——, boy, æt 11 years. October 1st, 1871. Comes to-day for first time, has had Otorrhœa since 5 years of age, which has continued till now, with a few short periods of improvement, the flow is free at present, hears only with difficulty. Exploration shows large perforations of "membrana tympani" in each ear, and intense inflammation of the membranes lining the cavity of middle ear, and large quantities of thick pus upon its surfaces. As the pus was so adherent, used the syringe with milk-warm water, and afterwards thoroughly dried the parts. Filled the ears with alcohol, after using "Douche," and kept the fluid in for some moments—slight pain, but soon followed by a very pleasant sensation. Patient has followed this treatment three times daily, returning to the Clinic once each week, up to this day, November 12th. Discharge has entirely disappeared, and only slight inflammation of the membranes of the cavity of the drum, remaining behind. Hearing much increased, cannot be made better, except by artificial means, as the natural memb. tymp. of both sides, are largely destroyed. To continue the medicine for one or two weeks longer, in order to reduce the remaining slight inflammation. Discharged.

